# Topical Delivery of Geranium/Calendula Essential Oil-Entrapped Ethanolic Lipid Vesicular Cream to Combat Skin Aging

**DOI:** 10.1155/2021/4593759

**Published:** 2021-09-11

**Authors:** Alka Lohani, Anurag Verma, G. Hema, Kamla Pathak

**Affiliations:** ^1^School of Pharmaceutical Sciences, IFTM University, 244102, Moradabad, India; ^2^Teerthanker Mahaveer College of Pharmacy, Teerthanker Mahaveer University, 244102, Moradabad, India; ^3^Department of Biotechnology, Maharani's Science College for Women, 560001, Bangalore, India; ^4^Pharmacy College Saifai, Uttar Pradesh University of Medical Sciences, 206130, Uttar Pradesh, India

## Abstract

The present study deals with the evaluation of the age-defying potential of topical cream formulations bearing Geranium essential oil/Calendula essential oil-entrapped ethanolic lipid vesicles (ELVs). Two types of cream formulations were prepared, viz., conventional and ELVs spiked o/w creams. Essential oil- (EO-) loaded ELVs were characterized by vesicle size, polydispersity index, encapsulation efficiency, and scanning electron microscopy. The cream formulations were evaluated for homogeneity, spreadability, viscosity, pH, *in vitro* antioxidant capacity, sun protection factor, and *in vitro* collagenase and elastase inhibition capacity. Confocal laser scanning microscopy (CLSM) was performed to ascertain skin permeation of conventional and vesicular cream. The results of *in vitro* antioxidant studies showed that GEO-/CEO-loaded vesicular creams have notable antioxidant capacity when compared to nonvesicular creams. GEO- or CEO-loaded vesicular creams exhibited the highest SPF value 10.26 and 18.54, respectively. Both the EO-based vesicular creams showed *in vitro* collagenase and elastase enzyme inhibition capacity. CLSM images clearly depicted that vesicular cream deep into the skin layers. From the research findings, the age-defying potential and photoprotective effects of GEO and CEO were confirmed. It can be concluded that ELVs are able to preserve the efficiency of EOs and have the potential to combat skin aging.

## 1. Introduction

Finding solutions against various signs of skin aging has been a natural human desire for centuries. Skin aging is a complex biological process, influenced by a combination of intrinsic (genetics, cellular metabolism, hormone, and metabolic processes) and extrinsic factors (chronic light exposure, pollution, ionizing radiation, chemicals, and toxins) [1]. These triggers cause the skin to deteriorate over a timeframe, affecting the wellbeing, wellness, and physical appearance of a person. Because of the fact that skin health and beauty are considered among the principal factors representing overall well-being in humans, age-defying cosmetic product market is observed to be one of the rising markets in today's world. Age-defying cosmetics can act efficaciously when they reach their target sites present in the deeper layers of the skin, but the stratum corneum is the biggest obstacle in delivering the actives deep into the skin layers. Several antiaging strategies have been developed during the past years to overcome this barrier. One of the possibilities for increasing the penetration of active ingredients is the use of vesicular delivery systems such as liposomes, niosomes, and ethosomes [2]. These vesicles can act either as a carrier system or as penetration enhancers. Lipid vesicles indicate their potential as carriers of cosmetics for plant extracts, phytochemicals, and other active ingredients which are poorly soluble, poorly absorbed, and unstable constituents. Lipid vesicles have shown tremendous potential to improve the effectiveness and efficiency of the delivery of cosmeceuticals and bioactive compounds [3]. Conventional liposomes, on the other hand, appear to be limited to the upper layers of the skin and act as a local reservoir for active ingredients with very low permeation into deeper skin layers [4]. Therefore, many strategies have been proposed to overcome the disadvantages of liposome vesicles. One of the interesting approaches is the use of ethanolic lipid vesicle. ELVs consist of phospholipid, ethanol, and water. The presence of ethanol in these vesicles makes the vesicular membrane highly flexible and malleable, and due to the solvent effect of ethanol, fluidity of stratum corneum lipids increases that leads to enhanced permeability of active ingredient.

One of the widely used plant extracts in cosmetics is essentials oils (EOs). EO is the complex mixtures containing dozens of substances of varying chemical compositions at different concentrations. They are a very important part of the perfume and cosmetic industry, but in the present scenario, their use is not limited to being used as fragrances only. EOs confer several benefits including antifungal [5], antibacterial [6], and antiviral properties [7], and most of these oils also boast powerful antioxidant benefits, which means they have the power to scavenge free radicals to protect the skin from damage [8]. However, their components are labile and volatile, and the sensory perception can be changed as a consequence of oxidation, heating, volatilization, or chemical interactions. These chemical and physical effects, which can alter the quality of products, can be effectively minimized by encapsulating the essentials oils.

In the present work, an attempt has been made to encapsulate Geranium/Calendula essential oil(s) in ethanolic lipid vesicles to prevent their evaporation and to increase their availability and efficacy in cosmetic products to combat skin aging.

## 2. Material

LipoidS-75 was obtained as a gift sample from Lipoid GmbH (Ludwigshafen, Germany). 2,2-Diphenyl-1-picryl-hydrazyl (DPPH) was obtained as a gift sample from HiMedia Laboratories, Mumbai. Coconut oil, olive oil, naphthylethylene diamine dihydrochloride, sodium nitroprusside, and sulphanilamide were procured from Sigma-Aldrich Chemical Pvt. Limited, Bangalore. Surfactants (tween 60 and span 60), ascorbic acid, geraniol, *α*-pinene, HPLC grade acetonitrile, water, and methanol were obtained from Central Drug House (P) Ltd., New Delhi. All the chemicals used were of analytical grade. The reagents were prepared using double distilled water.

## 3. Methods

### 3.1. Extraction of Essential Oils

In our previous work, we have reported extraction of Geranium essential oil (GEO) from *Pelargonium graveolens* leaves and Calendula essential oil (CEO) from *Calendula officinalis* flowers using a Clevenger apparatus. The extracted EOs were subjected to GC-MS (Gas Chromatography-Mass Spectrometry) analysis to get information about their chemical composition. Citronellol and geraniol were found to be the highly abundant chemical constituents of the GEO. The eminently ample chemical constituents present in CEO were *trans*-*β*-ocimene, dihydrotagetone, *cis*-tagetone, neo-allo-ocimene, 1,8 cineole, and *α*-pinene. Our findings showed that GEO and CEO have the potential to reduce or prevent oxidative stress and can be used in skincare regimens to slow down skin aging via its antioxidant properties [9].

### 3.2. Ethics Declaration

The present investigation was conducted according to the ethical principles and was approved by the Institutional Animal Ethical Committee, School of Pharmaceutical Sciences, IFTM University Moradabad, India (Registration No. 837/ac/04/CPCSEA).

### 3.3. Formulation of Ethanolic Lipid Vesicles (ELVs)

#### 3.3.1. Preliminary Optimization Studies

Preliminary studies were done to optimize the methodology. Cold method (described later) was used to prepare blank ELVs by varying the concentration of Lipoid S-75 (LS75) in the range of 0.5%*w*/*v*to 5.0%*w*/*v*and ethanol in the range of 10-40% *v*/*v* and analyzed by photomicrographs taken through optical photomicrograph (HICON, Delhi, India) at 100x.

#### 3.3.2. Preparation of Ethanolic Lipid Vesicles

ELVs were prepared by the cold method [5], composed of LS75 (2-3% *w*/*v*), ethanol (20-30% *v*/*v*), and propylene glycol (PG) (10% *v*/*v*). LS75 was dissolved along with the EO in ethanol. This mixture was heated to 30°C ± 1°C, and a fine stream of distilled water was added slowly, with constant mixing at 1000 rpm with a mechanical stirrer in a closed container. The preparation was left to cool at room temperature for 30 min, and then, it was sonicated at 4°C for two cycles of 2 min each with a minute rest between cycles using a sonicator. Various ELV formulations were prepared by varying the concentration of LS75, ethanol, and oil (Table 1).

### 3.4. Characterization of Essential Oil-Loaded ELVs

#### 3.4.1. Vesicle Size Measurement

The vesicle size and polydispersity index (PDI) of vesicular colloidal suspension were analyzed by a dynamic light scattering technique with Malvern Zetasizer Nano-ZS, Malvern, U.K. with DTS (Nano) software set at an angle of 173°. For vesicle size measurement, the vesicular suspension was diluted with distilled water (1 : 10) and put into the cuvettes of Malvern Zetasizer. Then, the measurements were conducted at 25°C.

#### 3.4.2. Encapsulation Efficiency

Geraniol and *α*-pinene, one of the major components of GEO and CEO, respectively, were chosen as an index for the determination of encapsulation efficacy (EE). The encapsulation of geraniol and *α*-pinene was measured by HPLC analysis. The vesicular suspension was transferred into a centrifuge tube and centrifuged for one hour at 30,000 rpm at 4°C using a cooling centrifuge (R-4C, Remi centrifuge, Vasai, India). After centrifugation, the supernatant and sediment were recovered and their volume was measured. Then, the supernatant was lysed using ACN : water (85 : 15) and filtered through a nylon filter disc (0.22 *μ*m). The index constituent was assayed both in the sediment and in the supernatant using HPLC to determine the EE [10].

(1) $$\begin{array}{c} \%\text{Encapsulation efficiency}=\frac{\text{Ct}-\text{Cs}}{\text{Cs}}\times 100. \end{array}$$

Ct is the total amount of oil detected both in supernatant and sediment; Cs is the amount of oil detected in supernatant. The EE was determined in triplicate.

#### 3.4.3. Scanning Electron Microscopy (SEM)

Optimized ELVs were visualized using a scanning electron microscope (Hitachi-H7500). A drop of 1% aqueous solution of phosphotungstic acid was added and left in contact with the sample for 5 min. The surplus solution was removed, and the sample was dried at room temperature, and then, the ELVs were viewed under SEM operating at an acceleration voltage of 80 kV.

### 3.5. Preparation of Cream Formulations

#### 3.5.1. Base Cream

The cream formulation was prepared by the phase inversion technique [11]. The cream composition is given in Table 2. The composition and amount of emulsifying agents were calculated by the HLB method. First of all, the oil constituents like cetyl alcohol, stearic acid, coconut oil, olive oil, and span-60 were mixed in a magnetic stirrer at 100 rpm at 60°C. The aqueous phase contained aloe vera gel and tween 60 as an emulsifying agent. The aqueous phase was added to the oil phase at 60°C with continuous mixing. When the mixture temperature reduced to 50°C, phase inversion took place and the viscosity of the emulsion was increased.

#### 3.5.2. Essential Oil-Loaded Vesicular/Nonvesicular Cream

The constituents and procedure for vesicular cream preparation were the same as those for the base cream, but while preparing vesicular cream, care was taken and the oil-loaded vesicles were added at a temperature below 30°C. In the preparation of nonvesicular cream, EO was added as such without encapsulation into the ELVs.

### 3.6. Characterization of Cream Formulations

#### 3.6.1. Physical Characterization

All the prepared cream formulations were characterized for color, odor, phase separation, and grittiness by visual observation. A small quantity of cream formulation was pressed between the thumb and index finger. The consistency of the cream was noticed (whether homogeneous or not), if there were any coarse particles that appeared on the fingers. Also, the homogeneity was also detected by rubbing a small quantity of cream on the skin back of the hand. The grittiness was also observed in the same manner.

#### 3.6.2. Spreadability, pH, and Viscosity

The spreadability of cream formulations was calculated by an apparatus suggested by Multimer [12] which is modified accordingly and used for the spreadability study. For the measurement of pH, cream formulations were diluted with distilled water in the ratio of 1 : 10 (cream : water) and mixed properly and their pH was measured by using a digital pH meter [13]. The viscosity of prepared cream formulations was measured by a Brookfield viscometer using T-spindle S-93 at 20 rpm. The temperature was maintained at 25°C ± 1°C. All the procedure was repeated three times, and observations are recorded as mean.

#### 3.6.3. Determination of Percent Essential Oil Content

The percentage content of oil present in the cream was determined by taking 10 mg of the cream and diluting it to 10 ml with the suitable solvent (ACN : water; 85 : 15). The sample was mixed by using a vortex shaker for 40 min and examined by HPLC to determine the percentage of oil present in the cream by measuring the index constituent(s).

#### 3.6.4. Stability Studies of Cream Formulations

The stability of the cream formulation(s) was assessed by storing the formulation at different storage conditions, namely, 8 ± 2°C, room temperature (25-28°C) and at 40 ± 2°C. The physical attributes (color, look, and feel), organoleptic parameters (phase separation, and liquefaction), pH, viscosity, spreadability, and oil content were also observed at various intervals for 30 days [14, 15].

### 3.7. Determination of Antioxidant Capacity

The antioxidant capacity of GEO- and CEO-based vesicular and nonvesicular cream formulations was determined by the following methods:

*DPPH radical scavenging capacity*: different dilutions of standard antioxidant (ascorbic acid) and cream formulation were prepared (10-250 *μ*g/ml) in methanol. DPPH solution in methanol (0.1 mM) was added to the equal volume of different dilutions of the sample and standard antioxidant. All the tubes were incubated (30°C) for 30 min in the dark. The absorbance of each solution was measured at 517 nm using a UV-visible spectrophotometer [9].

*Nitric oxide scavenging capacity*: sodium nitroprusside solution (10 mM) in phosphate buffer (pH 7.4) was added to the different dilutions (10-250 *μ*g/ml) of sample and standard (ascorbic acid) in methanol. The tubes were incubated (25°C) for 2 hrs. After that, 0.5 ml Griess reagent was added to the incubated tubes and absorbance was measured at 546 nm using a UV-visible spectrophotometer [9].

DPPH and nitric oxide radical scavenging capability was calculated by using following equation:

(2) $$\begin{array}{c} \%\text{Inhibition}=\frac{\text{Ac}-\text{As}}{\text{Ac}}\times 100, \end{array}$$

where Ac is the absorbance of control and As is absorbance of cream sample/standard.

A linear regression equation was obtained by plotting percent inhibition on the*y*-axis and concentration (*μ*g/ml) on the *x*-axis in a graph, and from this equation, the IC_50_ value was calculated.

Percent inhibition of each sample dilution was plotted by taking on the *y*-axis and concentration (*μ*g/ml) on the *x*-axis in a graph to obtain a linear regression equation, and from this equation, the IC_50_ value (concentration of the sample required to scavenge 50% free radical.) was calculated. The experiment was done in triplicate.

### 3.8. Determination of Sun Protection Factor (SPF) of Cream Formulations

For practical, economical, and ethical reasons, the *in vitro* SPF measurement techniques represent an acceptable and speedy tool for shortening *in vivo* risks and experiments related to UV exposure of human subjects. *In vitro* SPF of cream formulations was determined as per the COLIPA standards [16] which include the measurement of the percent transmittance of a sunscreen product across the UV spectrum weighted by the erythemal weighting factors at different wavelengths [17].

(3) $$\begin{array}{c} {\text{SPF}}_{\text{spectrophotometric}}=\text{CF}\times \overset{320}{\underset{290}{\sum }}\text{E}\text{E}\left(\lambda \right)\times I\left(\lambda \right)\times \text{Abs}\left(\lambda \right), \end{array}$$

where CF is correction factor (10), EE(*λ*) is erythmogenic effect of radiation with wavelength *λ*, and Abs (*λ*) is spectrophotometric absorbance values at wavelength *λ*. The values of EE (*λ*) × *I* are constants.

### 3.9. *In Vitro* Enzyme Inhibition Assay

After getting the results from *in vitro* antioxidant assays and SPF determination, optimized cream formulations (GC3 and CC3) were selected for the *in vitro* enzyme inhibition assay.

#### 3.9.1. In Vitro Collagenase Inhibition Assay

For this assay, 1, 10, 50, 100, 500, and 1000 *μ*g/ml concentration of selected cream samples in ethanol was prepared. 5 *μ*l of each concentration of test sample was taken in a reaction mixture along with enzyme in total volume of 80 *μ*l, and the reaction mixture was incubated for 15 minutes. After incubation, 20 *μ*l of the substrate (FALGPA) was added to each reaction mixture and readings were recorded at 345 nm/660 nm for 10 min at 1 min interval. Epigallocatechin gallate (EGCG) was taken as positive control.

#### 3.9.2. In Vitro Elastase Inhibition Assay

In this assay, 1, 10, 50, 100, 500, and 1000 *μ*g/ml concentration of selected cream samples in ethanol was prepared. 5 *μ*l of each concentration was taken in reaction mixture along with elastase enzyme in total volume of 90 *μ*l, and the reaction mixture was incubated for 15 minutes. After incubation, 10 *μ*l of the substrate (N-succinyl-Ala-Ala-Ala-p-nitroanilide) was added to each reaction mixture and the readings were recorded at 405 nm/660 nm for 10 min at 1 min interval. Epigallocatechin gallate (EGCG) was taken as positive control.

In both the enzyme inhibition assays, the percent inhibition was calculated by using the following equation:

(4) $$\begin{array}{c} \%\text{Inhibition}=\frac{\text{AbC}-\text{AbS}}{\text{AbC}}\times 100, \end{array}$$

where AbC is absorbance of control and AbS is absorbance of sample/standard.

### 3.10. Skin Irritation Study

Albino rats of either sex, weighing 150-180 g, were used for skin irritation study (*n* = 3 in each group). The animals were divided into two groups, namely, the controlled and test groups. Before three days of starting the study, hair was shaved from the back of rats and a 5 cm^2^ area was marked. The cream formulations were applied to the marked site, and the site was observed for any reaction or sensitivity and slight/moderate or severe erythema till 3 days after application. To score the skin reactions for erythema, scar, and edema, the Draize skin irritation scoring system was selected [18]. The reactions, defined as erythema and edema, were evaluated according to the scoring system for skin reactions. The score of primary irritation (SPI) was calculated for each rat. Scores for erythema and edema at 24, 48, and 72, hours were summed and divided by the number of the observations for the treated sites. The SPI for the control sites were calculated in the same fashion as the test.

### 3.11. Confocal Laser Scanning Microscopy (CLSM) Study

Skin permeation depth and mechanism of rhodamine red-loaded vesicles were examined by CLSM. Rhodamine-loaded vesicular cream was formulated by adding the dye to the mixture of LS75 in ethanol and PG, and the prepared dye-loaded vesicles were incorporated into the cream base. Dye-loaded vesicular and the nonvesicular cream formulation was applied to the dorsal rat skin for 8 h. The rats were sacrificed, and the skin was excised and washed. The skin sections were prepared and examined with CLSM (Fluoview FV 1000, Olympus, Japan) [19].

## 4. Results and Discussion

### 4.1. Formulation of ELVs

Phospholipid and ethanol are the basic materials composing ELVs and play an important role in vesicle characteristics such as size, entrapment efficacy, and stability. The phospholipid is responsible for the formulation of a lipid bilayer that affects the stability of the vesicle and also enhances the rigidity and prevents leakage of the enclosed material. The optimum concentration of ethanol enhances the membrane elasticity and fluidity of vesicles that may contribute to high skin permeability.

**Figure 1:**
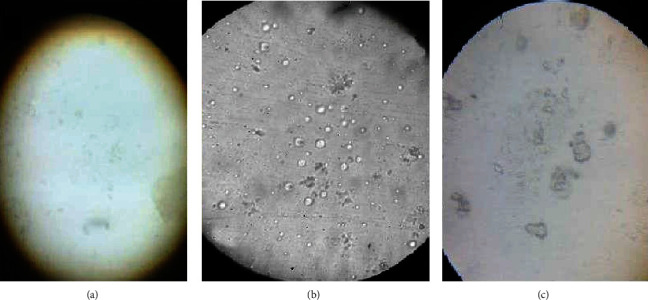
Optical microscopic image of prepared vesicles at different concentrations of LS75: (a) below 2% *w*/*v* (vesicles could not form); (b) 2-4% *w*/v (spherical vesicles with well-defined boundaries); (c) above 4% *w*/*v* (larger vesicles with irregular shape).

Preliminary screening studies were carried out to identify the effect of variables that influence the physicochemical properties of vesicles and to optimize the methodology. Placebo (blank) ELVs were prepared by using varied concentration of LS75 (0.5%-4.0% *w*/*v*) and ethanol (10-40% *v*/*v*). The results showed that below 2% *w*/*v* concentration of LS75, the vesicles could not form (Figure 1(a)). It was observed that between 2-4% *w*/*v* of LS75, the vesicles were uniformly distributed with spherical shape and well-defined boundaries (Figure 1(b)) and above 4% *w*/*v* larger vesicles were formed with irregular shape (Figure 1(c)). ELVs were formulated by using LS75 in the concentration range of 2-4% *w*/v and ethanol in the range of 30-40% *v*/*v*.

### 4.2. Characterization of Essential Oil-Loaded ELVs

#### 4.2.1. Vesicle Size Measurement

**Figure 2:**
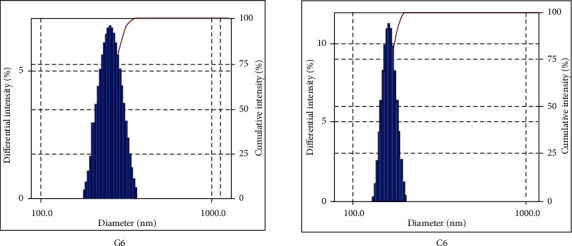
Vesicle size distribution diagram of optimized ethanolic lipid vesicles (G6 and C6).

Vesicle size is one of the important parameters that affect the permeability across the skin. Developed EO-loaded ELVs were varied in the size range of 192.0 nm-543.1 nm (Table 3). The vesicle size distribution diagram of optimized formulations (G6 and C6) is shown in Figure 2. The results showed that vesicle size was directly proportional to the concentration of LS75 and indirectly proportional to the concentration of ethanol [20], i.e., as the concentration of LS75 was increased, vesicle size was also increased and upon increasing the concentration of ethanol, the size of vesicles got reduced. It has been reported in the previous studies that a high concentration of ethanol leads to the interpenetration of the ethanol hydrocarbon chain, which results in slimming down of vesicle membrane thickness and hence causes a reduction in vesicle size. Some researchers have suggested that this may be due to the fact that high concentration of ethanol modifies the vesicular surface characteristics and alters the net charge, which could lead to a decrease in mean vesicle size [21].

#### 4.2.2. Polydispersity Index (PDI)

PDI number is a description of the dispersion of size populations within a given sample. The stability of vesicular formulation depends upon the homogenous populations of vesicles of a certain size. PDI number ranges from 0.0 to 1.0. The best PDI value is 0.0 which indicates homogenous dispersion with respect to the vesicle size, and the PDI value 1.0 indicates an extremely polydisperse sample with multiple vesicular size populations [22]. In the case of lipid-based vesicular carriers, a PDI value of 0.3 and below is considered to be agreeable and shows a homogenous dispersion of lipid vesicles [23, 24]. The results showed that all the vesicular formulations have a PDI value less than 1.0 and ranged from 0.045 to 0.301, which indicates that vesicles are homogenously distributed (Table 3).

#### 4.2.3. Encapsulation Efficiency

EE of vesicular formulations is the part of total oil entrapped in the prepared vesicles, which determines the oil holding capacity and ultimately the delivery potential to the particular site. The EE was found to be in the range from 58.92 ± 0.33% to 90.95 ± 0.29% (Table 3). Results showed that both the amount of LS75 and the concentration of ethanol influence the encapsulation of oil in vesicles positively. It was noticed that with the increase in the concentration of LS75 from 2% to 4% and ethanol from 20% to 30% ,the entrapment of oil inside the vesicles also increased. Based on the physicochemical characterization, the optimized vesicular formulations G6 (GEO encapsulated) and C6 (CEO encapsulated) were selected for visualization.

#### 4.2.4. Scanning Electron Microscopy (SEM)

**Figure 3:**
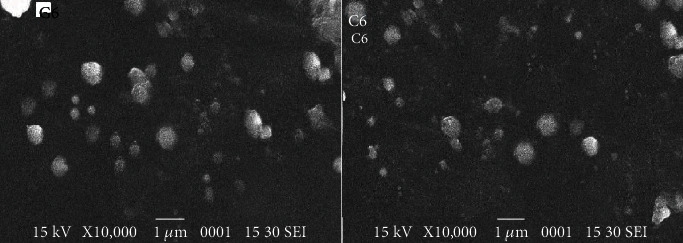
SEM image of optimized ethanolic lipid vesicles: G6 (GEO encapsulated) and C6 (CEO encapsulated).

SEM results of optimized ethanolic lipid vesicles loaded with GEO and CEO, i.e., G6 and C6, respectively, disclosed the dominance of spherical vesicular carriers as shown in Figure 3.

### 4.3. Selection of Optimized ELVs

Based on the results obtained from the characterization of ELVs, G6 (GEO encapsulated) and C6 (CEO encapsulated) were selected for incorporation into the cream base.

### 4.4. Characterization of Cream Formulations

#### 4.4.1. Physical Characterization

All the cream formulations were white in color with a mild characteristic odor of the EO used. The prepared cream formulations were homogeneous with a complete absence of lumps and grittiness.

#### 4.4.2. Spreadability, Viscosity, and pH

A cream should not generate friction while applied on the skin and should spread easily. Spreading quality of cream helps in the uniform application to the skin. The spreadability result of prepared creams was found in the range of 17.68 ± 0.34 gm·cm/sec to 25.50 ± 0.45 gm·cm/sec. The results express the ability of the creams to spread on the application of a small amount of shear. The spreadability characteristics are also influenced by viscosity. The viscosity of the cream formulations ranged between 2713.5 ± 1.02 and 6011.2 ± 1.20 cp which indicate substantial consistency. Furthermore, the pH values of all developed formulations were in the range 6.8 ± 0.052 gm·cm/sec to 7.1 ± 0.050 gm·cm/sec (Table 4). The pH values lie in the normal pH range of the skin and would not produce irritation upon application to the skin.

#### 4.4.3. Determination of Percent Essential Oil Content

The EO content determination results indicated that the EO was uniformly distributed throughout the vesicular cream formulation. It was interesting to observe that in the case of free EO-loaded creams, the EO content results were significantly reduced; this might be due to the loss of free EO during the cream formulation process (Table 4).

### 4.5. Stability Studies of Cream Formulations

The motive behind stability testing of a cosmetic product is to confirm that the tested product meets the intended chemical and physical quality standards, functionality, and aesthetics when stored under suitable storage conditions. The freshly prepared creams were white in color, but a slight change in color was observed for GC1 formulation after 30 days and for CC1 after 21 days when stored at 40°C; this may be due to the separation of the oil phase at high temperature. No sign of liquefaction was observed in the tested creams at different storage conditions for 30 days of observation. There was no phase separation in any tested creams after centrifugation and freeze and thaw test. It was observed that the freezing lowered the viscosity of tested creams [25]. The absence of any sign of liquefaction and phase separation provided strong evidence for the stability of the creams under investigation.

There was no significant change in the pH value of tested creams at various storage conditions. The oil content in creams decreased at higher temperature suggesting storage at room temperature or cool place. None of the stability parameters changed significantly at room temperature.

### 4.6. Determination of Antioxidant Capacity

Antioxidants are capable to guard against skin damage and slow down the skin aging process. The antioxidant capacities of extracted GEO and CEO have been previously reported by us [9], and their assessment of the antioxidant capacity after formulation as ELV-based cream was carried out.

#### 4.6.1. Nitric Oxide Scavenging Capacity

**Figure 4:**
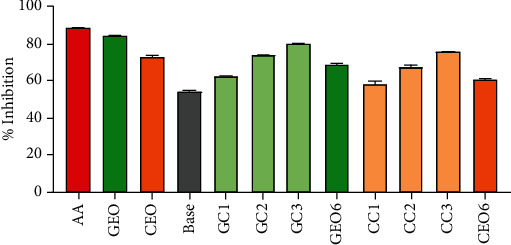
Comparison of nitric oxide scavenging capacity of standard (AA); essential oils (GEO and CEO); base cream; GEO-loaded vesicular creams (GC1, GC2, and GC3); CEO-loaded vesicular creams (CC1, CC2, and CC3); and nonvesicular cream GEO6 and CEO6 for GEO and CEO, respectively.

Sodium nitroprusside decomposes in an aqueous physiological solution (at pH 7.4) results in the generation of nitric oxide. Nitric oxide is a free radical that reacts with oxygen under aerobic conditions and generates nitrite ions. The principle of this scavenging technique relies on the measurement of the capacity of the antioxidant to trap nitric oxide, leading to a decreased production of nitrite ions. The results of nitric oxide scavenging capacity are shown in Figure 4. The nitric oxide scavenging capacity of the standard (ascorbic acid) was 89.19 ± 0.05% at 250 *μ*g/ml. The nitric oxide scavenging capacity of extracted EOs was previously reported by us as 85.15 ± 0.09% and 72.48 ± 0.12% for GEO and CEO, respectively [9]. In the case of GEO-/CEO-loaded vesicular and nonvesicular cream formulations (Figure 3), the maximum inhibition was shown by GC3 (80.96 ± 0.20%) and CC3 (75.21 ± 0.31%) formulation for GEO and CEO, respectively.

#### 4.6.2. DPPH Scavenging Capacity

In this method, the degree of DPPH radical discoloration is often used as an index of the antioxidant capacity of the tested samples. In our previous work [9], we observed that GEO has a higher power to diminish the dark violet color of DPPH radical to yellow diphenylpicrylhydrazine radical in comparison to CEO and that GEO was found to be rich in monoterpenoid and citronellol and geraniol were the major component of the oil and they also have been previously identified as a potential antioxidant [26, 27]. The DPPH radical scavenging capacity of extracted EOs was previously reported by us as 85.51 ± 0.020% (for GEO) and 78.06 ± 0.04% (for CEO) at 250 *μ*g/ml [9].

**Figure 5:**
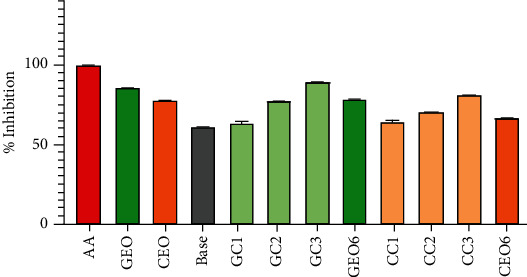
Comparison of DPPH radical scavenging capacity of standard (AA); essential oils (GEO and CEO); base cream; GEO-loaded vesicular creams (GC1, GC2, and GC3); CEO-loaded vesicular creams (CC1, CC2, and CC3); and nonvesicular cream GEO6 and CEO6 for GEO and CEO, respectively.

The highest DPPH radical scavenging capacity (99.08 ± 0.08%) was obtained by standard ascorbic acid in the concentration of 250 *μ*g/ml. At the same concentration, the maximum percent inhibition was shown by GC3 (89.82 ± 0.11%) and CC3 (80.89 ± 1.03%) formulation, respectively (Figure 5). The DPPH radical scavenging capacities of EO-loaded vesicular cream formulations were found to be remarkable in comparison to standard antioxidant and EO alone.

The results of both the antioxidant assay showed that GEO- or CEO-loaded vesicular cream formulations have notable antioxidant capacity when compared to EO alone. The results were eye-catching when the antioxidant capacity of vesicular cream formulations was compared with the cream formulation containing the same concentration of unentrapped or free EO. It was interesting to observe that with both the EOs, the scavenging capacity was reduced for nonvesicular cream formulation in comparison to the same concentration of vesicular cream. This may be due to the instability or loss of EO during the formulation of nonvesicular cream formulation.

### 4.7. Determination of *In Vitro* Sun Protection Factor

**Figure 6:**
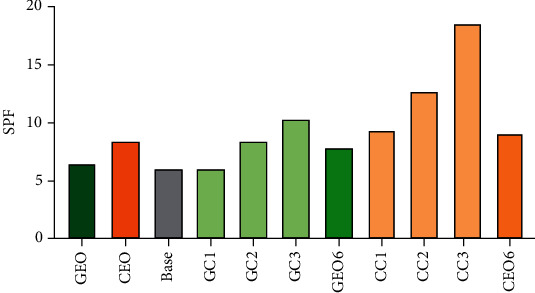
Comparison of *in vitro* SPF values of essential oils (GEO and CEO), base cream, GEO-loaded vesicular creams (GC1, GC2, and GC3), CEO-loaded vesicular creams (CC1, CC2, and CC3), and free oil-loaded nonvesicular cream GEO6 and CEO6 for GEO and CEO, respectively.

*In vitro* SPF determination is a useful test for screening ingredients during the development stage of a cosmetic product. The higher the SPF, the more the protection offered by sunscreen against UV light. An EO should absorb major UV radiations (290-400 nm) to be adequately used in cosmetic formulations to prevent photoaging, sunburn, skin wrinkles, and other skin damages. In our previous work, the SPF value of GEO and CEO was found to be 6.45 and 8.36, respectively [9]. EO-based vesicular cream formulations showed remarkably high SPF in comparison to EO alone. The SPF value of GEO-loaded vesicular cream was found to be 6.02, 8.35, and 10.26 for GC1, GC2, and GC3, respectively (Figure 6). The SPF of CEO-loaded vesicular cream was found to be 9.28, 12.68, and 18.54 for CC1, CC2, and CC3, respectively. The SPF of free EO-loaded nonvesicular cream formulation was 7.82 (for GEO) and 9.02 (for CEO) which is very low in comparison to the vesicular cream at the same concentration of EO. The overall results showed that CEO-based cream formulation had higher SPF in comparison to GEO-based cream formulation.

### 4.8. *In Vitro* Enzyme Inhibition Assay

One of the main reasons behind various signs of skin aging is the damage and loss of two key proteins: collagen and elastin [28]. These proteins are essential for maintaining youthful skin, which are found in the deeper layers of the skin. Collagen plays a major role in skin strengthening, and elastin allows the body tissues to return to their original shape either by contracting or stretching. These proteins are essential for skin and work together to create skin firmness and help to hold the skin shape and strength [29, 30]. The main cause of degradation of these proteins is matrix metalloproteinases (MMPs). MMP enzymes such as collagenase and elastase are responsible for the breakdown of collagen and elastin protein, respectively. Degradation of the two essential skin proteins by these enzymes accelerates skin wrinkling and sagging skin appearance and leads to skin aging. Thus, it is important to determine the enzyme inhibiting activity of the developed formulations in order to assess the antiaging potential. This is the first-time research to investigate the antielastase, anticollagenase potential of GEO-/CEO-based formulation. The optimized cream formulations (GC3 and CC3) from each EO were used for further investigation of enzyme inhibition assay.

#### 4.8.1. In Vitro Collagenase Inhibition Assay

**Figure 7:**
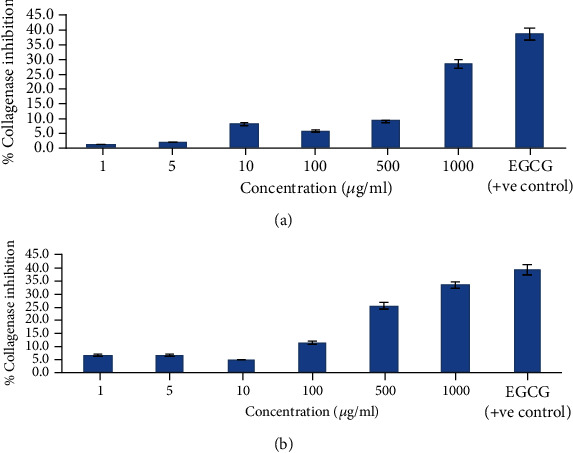
*In vitro* collagenase inhibition assay: (a) % collagenase inhibition shown by GEO-loaded optimized cream formulation GC3; (b) % collagenase inhibition shown by CEO-loaded optimized cream formulation CC3.

The assay is based on an enzyme-substrate interaction, i.e., between collagenase enzyme and the synthetic collagen substrate N-[3-(2-furyl) acryloyl]-Leu-Gly-Pro-Ala (FALGPA). Proteolytic degradation of this collagen substrate due to the collagenase enzyme results in a decrease in absorbance. Collagenase inhibitors prevent the degradation of FALGPA [31]. The EOs incorporated in the creams have potential for collagenase inhibition that was assessed by the assay. From the results of the collagenase inhibition assay, it was observed that both the formulations showed anticollagenase activity in a concentration-dependent manner (Figure 7). It was observed that the anticollagenase activity increases as the concentration increases from 1 to 100 *μ*g/ml. In the case of GC3, formulation maximum inhibition (28.4%) was observed at 1000 *μ*g/ml, whereas at the same concentration, CC3 showed the maximum inhibition of 22.9%.

#### 4.8.2. In Vitro Elastase Inhibition Assay

**Figure 8:**
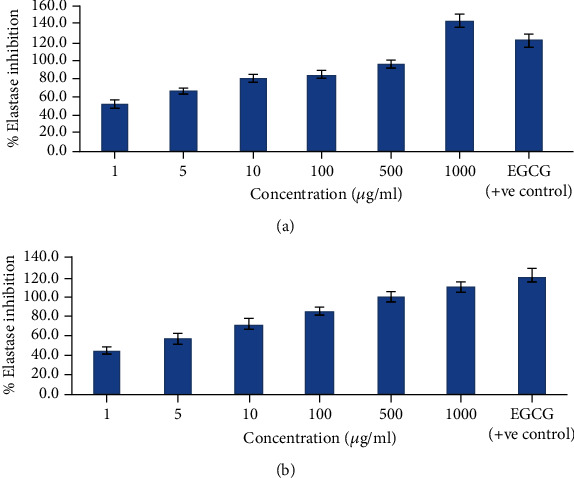
*In vitro* elastase inhibition assay: (a) % elastase inhibition shown by GEO-loaded optimized cream formulation GC3; (b) % elastase inhibition shown by CEO-loaded optimized cream formulation CC3.

The elastase inhibition assay is based on the fact that the enzyme (elastase) causes the breakdown of the elastin substrate (N-succinyl-Ala-Ala-Ala-p-nitroanilide) and yields fluorescent fragments that are measured using a fluorescence microplate reader. This enzymatic hydrolysis is interrupted by the elastase inhibitors [32]. The elastase inhibition activity of the developed creams was assessed to determine their antiaging potential. The antielastase activities of both the optimized cream formulations (GC3 and CC3) are shown in Figure 8. The antielastase activity was found to be in a concentration-dependent manner. In the case of GEO-loaded vesicular cream formulation (GC3), it was surprising to see that the antielastase inhibition was higher (144.0%) than ECGC (122.4%) at the concentration of 1000 *μ*g/ml. CEO-loaded vesicular cream formulation (CC3) also showed significant elastase inhibition activity. The highest antielastase inhibition was observed at 108.2% at the same concentration i.e., 1000 *μ*g/ml.

The findings have verified that the GEO-/CEO-loaded vesicular creams may contribute to the fight against skin aging by preventing collagen and elastin degradation underneath the skin and can help to restore skin strength and elasticity and thereby slow down the skin wrinkling process.

### 4.9. Skin Irritation Study

In this study, rats were divided into 5 groups (*n* = 3 in each group). In group 1, base cream was applied, in groups 2 and 3 optimized vesicular cream of each EO (GC3 and CC3), and in groups 4 and 5 nonvesicular cream formulation of each EO (GEO6 and CEO6). The results obtained from the primary skin irritation studies (Table 5) interpreted according to Draize test [17] which says that test samples that produce PII scores of 2 or less are considered negative, i.e., no skin irritation. Since, the score between 0 and 2 suggests no to mild irritation, thus, low PII of vesicular cream formulation (0.27 for GC3 and 0.16 for CC3 cream formulations) in comparison to base cream (0.16) observed in the study depicted nonirritancy of the cream formulation and could be considered safe for use, while the primary skin irritation studies of free EO-loaded nonvesicular cream showed high PII values (0.55 for GEO6 and 0.49 for CEO6 cream formulation) which indicates slight skin irritation in comparison to vesicle-based cream formulations of the same EO.

### 4.10. Confocal Laser Scanning Microscopy (CLSM) Study

**Figure 9:**
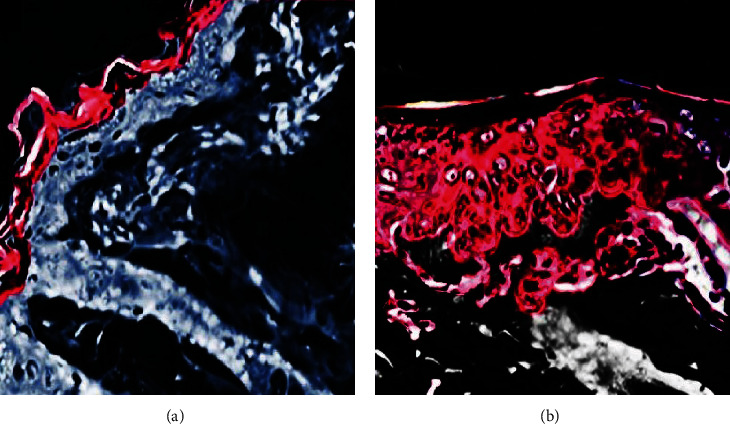
CLSM of (a) Rhodamine-loaded nonvesicular cream confined to the upper layer of the skin epidermis; (b) enhanced skin permeation observed by rhodamine-loaded vesicular cream.

Cream formulations loaded with and without lipid vesicles were prepared, and penetration across the skin was measured by CLSM study. Skin permeation of rhodamine (marker) from rhodamine-loaded nonvesicular cream and rhodamine-loaded vesicular cream formulation was visualized through a confocal laser scanning microscope. It was observed that permeation from rhodamine-loaded nonvesicular cream (Figure 9(a)) was confined only to the upper layer of the skin epidermis, while in the case of rhodamine-loaded vesicular cream (Figure 9(b)), enhanced permeation of rhodamine was observed deep into the skin layers. This shows that prepared ELVs have the ability to carry antiaging EO deep into the skin where the root cause of skin aging exists.

## 5. Conclusion

Finding solutions against the signs of skin aging has been a natural human desire for centuries. Because of this, the market is flooded with beauty care products claiming magical results within a short period of application. It is to be noted that natural aging is genetically determined, but extrinsic aging can be slowed down with the use of scientifically designed and evaluated cosmetic formulations. The need of the hour is to put serious efforts into the development of such products, which actually translate the stated claims in the case of extrinsic aging. From the research findings, the antioxidant potential, ability to inhibit collagenase and elastase enzyme, and photoprotective effects of GEO/CEO vesicular cream were confirmed. Results clearly showed that ELVs were able to preserve the efficiency of essential oils and have the potential to deliver the actives deeper into the skin. Essential oil-encapsulated vesicular creams were able to provide antioxidant defence mechanism with high SPF and are able to protect the skin in comparison to free essential oil-loaded nonvesicular creams. The cumulative effect of ELVs and cream composition and essential oil will collectively produce a protective effect to combat skin aging.

## Figures and Tables

**Table 1:** Composition of ethanolic lipid vesicles.

Formulation code	LipoidS-75 (% *w*/*v*)	GEO (% *w*/*v*)	CEO (% *w*/*v*)	Ethanol (% *v*/*v*)	Propylene glycol (% *v*/*v*)
G1	2	4	—	20	10
G2	3	4	—	20	10
G3	4	4	—	20	10
G4	2	4	—	30	10
G5	3	4	—	30	10
G6	4	4		30	10
C1	2	—	4	20	10
C2	3	—	4	20	10
C3	4	—	4	20	10
C4	2	—	4	30	10
C5	3	—	4	30	10
C6	4	—	4	30	10

**Table 2:** Composition of various cream formulations, i.e., base cream; vesicular cream (GEO loaded: GC1, GC2, and GC3 and CEO loaded: CC1, CC2, and CC3); nonvesicular cream GEO6 and CEO6 for GEO and CEO, respectively.

Ingredients (% *w*/*w*)	Base cream	GC1	GC2	GC3	GEO6	CC1	CC2	CC3	CEO6
Beeswax	8	8	8	8	8	8	8	8	8
Stearic acid	4	4	4	4	4	4	4	4	4
Cetyl alcohol	3	3	3	3	3	3	3	3	3
Olive oil	12	12	12	12	12	12	12	12	12
Coconut oil	16	16	16	16	16	16	16	16	16
Span60	1.4	1.4	1.4	1.4	1.4	1.4	1.4	1.4	1.4
Tween60	1.5	1.5	1.5	1.5	1.5	1.5	1.5	1.5	1.5
Aloe vera gel	qs	qs	qs	qs	qs	qs	qs	qs	qs
GEO loaded in vesicles	—	2	4	6	—	—	—	—	—
CEO loaded in vesicles	—	—	—	—	—	2	4	6	—
Free GEO	—	—	—	—	6	—	—	—	—
Free CEO	—	—	—	—	—	—	—	—	6

**Table 3:** Results of vesicle size, polydispersity index, and encapsulation efficiency of ethanolic lipid vesicles.

S. No.	Formulation code	Vesicle size (nm)	Polydispersity index (PDI)	Encapsulation efficiency (%)
1	G1	411.3	0.292	58.92 ± 0.33
2	G2	458.9	0.089	62.62 ± 0.64
3	G3	543.1	0.274	78.95 ± 0.69
4	G4	218.3	0.131	68.24 ± 0.87
5	G5	245.9	0.288	80.95 ± 0.49
6	G6	199.6	0.063	89.00 ± 0.85
7	C1	339.5	0.208	60.24 ± 0.78
8	C2	399.1	0.292	69.52 ± 1.64
9	C3	424.7	0.301	78.39 ± 0.61
10	C4	185.0	0.172	72.81 ± 0.46
11	C5	220.1	0.045	84.60 ± 0.82
12	C6	192.0	0.107	90.95 ± 0.29

**Table 4:** Characterization of GEO-/CEO-based vesicular and nonvesicular cream formulations.

Formulation code	Spreadability (gm·cm/sec)	pH	Viscosity (cp)	EO content (%)
Base cream	17.68 ± 0.34	6.8 ± 0.052	2713.5 ± 1.02	—
GC1	20.68 ± 0.56	7.0 ± 0.091	5413.5 ± 1.60	90.46 ± 1.98
GC2	22.82 ± 0.82	6.9 ± 0.056	5402.0 ± 1.58	93.78 ± 1.05
GC3	25.50 ± 0.45	6.9 ± 0.048	6011.2 ± 1.20	98.23 ± 1.60
CC1	18.06 ± 0.92	6.8 ± 0.060	5424.5 ± 2.60	95.62 ± 1.90
CC2	20.42 ± 0.42	7.1 ± 0.050	4302.5 ± 2.01	97.48 ± 2.01
CC3	22.49 ± 0.60	7.0 ± 0.078	4413.5 ± 1.98	98.66 ± 1.50
GEO6	20.60 ± 0.74	7.0 ± 0.028	2878.5 ± 1.02	74.78 ± 2.20
CEO6	18.68 ± 0.90	6.9 ± 0.040	4028.6 ± 1.00	71.89 ± 1.82

**(a):** 

Skin reaction	Time (hrs)	Group 1 (cream base)			Group 2 (formulation GC3)			Group 3 (formulation CC3)		
		Rat-1	Rat-2	Rat-3	Rat-1	Rat-2	Rat-3	Rat-1	Rat-2	Rat-3

Erythema	24	1	1	1	1	1	1	1	0	0
	48	0	0	0	0	0	0	0	0	0
	72	0	0	0	0	0	0	0	0	0

Edema	24	0	0	0	1	0	1	1	1	0
	48	0	0	0	0	0	0	0	0	0
	72	0	0	0	0	0	0	0	0	0

Score of primary irritation		0.16	0.16	0.16	0.33	0.16	0.33	0.33	0.16	0.0

Primary irritation index		0.16			0.27			0.16		

**(b):** 

Skin reaction	Time (hrs)	Group 4 (GEO6)			Group 5 (CEO6)		
		Rat-1	Rat-2	Rat-3	Rat-1	Rat-2	Rat-3

Erythema	24	1	1	1	1	1	1
	48	1	0	1	1	1	1
	72	0	0	0	0	0	0

Edema	24	1	1	1	1	1	0
	48	1	0	1	0	1	0
	72	0	0	0	0	0	0

Score of primary irritation		0.66	0.33	0.66	0.50	0.66	0.33

Primary irritation index		0.55			0.49		

## Data Availability

Data are available in Supplementary files. Supplementary data supporting the findings of this study includes the image of optimized cream formulations, detailed results of antioxidant capacity, and SPF values of prepared cream formulations.

## Supplementary Materials

Supplementary Materials Supplementary data supporting the findings of this study includes the image of optimized cream formulations, detailed results of antioxidant capacity, and SPF values of prepared cream formulations.
