# Supplementary material for: Topical Delivery of Geranium/Calendula Essential Oil-Entrapped Ethanolic Lipid Vesicular Cream to Combat Skin Aging
Source: Biomed Res Int. 2021 Sep 11;2021:4593759. doi: 10.1155/2021/4593759 (PMC8452390; doi:10.1155/2021/4593759)
Supplement: Supplementary Materials — Supplementary data supporting the findings of this study includes the image of optimized cream formulations, detailed results of antioxidant capacity, and SPF values of prepared cream formulations. [file 4593759.f1.docx]

**EXPERIMENTAL DATA**

**Prepared Optimized cream formulations**

**Table: Nitric Oxide Scavenging Capacity**

| Code | Ascorbic acid | GEO | CEO | Base Cream |
| --- | --- | --- | --- | --- |
| % Inhibition  (at 250 µg/ml) | 89.19±0.05 | 85.15±0.09 | 72.48±0.12 | 54.01±0.32 |
| IC_50 (_µg/ml) | 2.13 | 19.98 | 28.64 | 179.89 |
| Code | **GC1** | **GC2** | **GC3** | **GEOC6** |
| % Inhibition  (at 250 µg/ml) | 62.02±0.32 | 74.42±0.28 | 80.96±0.20 | 68.23±0.52 |
| IC_50 (_µg/ml) | 139.71 | 48.45 | 27.21 | 97.54 |
| Code | **CC1** | **CC2** | **CC3** | **CEOC6** |
| % Inhibition  (at 250 µg/ml) | 58.82±0.78 | 67.36±0.55 | 75.21±0.31 | 60.17±0.52 |
| IC_50 (_µg/ml) | 157.41 | 62.95 | 20.87 | 101.75 |

**Table: DPPH Scavenging Capacity**

| Code | Ascorbic acid | GEO | CEO | Base Cream |
| --- | --- | --- | --- | --- |
| % Inhibition  (at 250 µg/ml) | 99.08±0.08 | 85.51±0.10 | 78.06±0.11 | 60.89±0.10 |
| IC_50 (_µg/ml) | 10.38 | 18.02 | 28.68 | 91.64 |
| Code | **GC1** | **GC2** | **GC3** | **GEOC6** |
| % Inhibition  (at 250 µg/ml) | 64.91±0.12 | 77.64±0.11 | 89.82±0.11 | 78.67±0.10 |
| IC_50 (_µg/ml) | 90.92 | 47.83 | 10.11 | 48.64 |
| Code | **CC1** | **CC2** | **CC3** | **CEOC6** |
| % Inhibition  (at 250 µg/ml) | 64.87±1.02 | 70.46±1.00 | 80.89±1.03 | 66.57±1.01 |
| IC_50 (_µg/ml) | 158.04 | 73.19 | 24.28 | 87.12 |

**Table: SPF Value of GEO, CEO and Base cream**

| Wavelength  (λ_max_ nm) | Absorbance^*^ | | |
| --- | --- | --- | --- |
|  | **GEO** | **CEO** | **Base Cream** |
| 290 | 0.902±0.09 | 0.946±0.12 | 0.622±0.09 |
| 295 | 0.824±0.10 | 0.928±0.09 | 0.519±0.08 |
| 300 | 0.768±0.08 | 0.893±0.14 | 0.445±0.10 |
| 305 | 0.620±0.12 | 0.820±0.12 | 0.287±0.12 |
| 310 | 0.518±0.09 | 0.798±0.11 | 0.235±0.09 |
| 315 | 0.442±0.09 | 0.721±0.11 | 0.198±0.09 |
| 320 | 0.424±0.10 | 0.652±0.12 | 0.168±0.09 |
| Calculated SPF | 6.45 | 8.36 | 3.37 |

^*^ n=3±SEM

**Table: SPF Value of GEO Loaded Vesicular Cream Formulations**

| Wavelength  (λ_max_ nm) | Absorbance^*^ | | | |
| --- | --- | --- | --- | --- |
|  | **GC1** | **GC2** | **GC3** | **GEOC6** |
| 290 | 0.183±0.01 | 0.887±0.04 | 1.023±0.14 | 0.015±0.10 |
| 295 | 0.335±0.03 | 0.708±0.01 | 1.868±0.11 | 0.082±0.12 |
| 300 | 0.453±0.01 | 0.887±0.03 | 0.978±0.12 | 0.287±0.11 |
| 305 | 0.615±0.02 | 0.756±0.04 | 0.991±0.10 | 0.328±0.10 |
| 310 | 0.881±0.01 | 0.862±0.01 | 0.8953±0.12 | 0.186±0.11 |
| 315 | 0.796±0.01 | 0.987±0.02 | 0.8785±0.11 | 0.0837±0.09 |
| 320 | 0.538±0.02 | 0.978±0.02 | 0.6789±0.11 | 0.018±0.11 |
| Calculated SPF | 6.02 | 8.35 | 10.26 | 7.82 |

^*^ n=3±SD

**Table: SPF Value of CEO Loaded Vesicular Cream Formulations**

| Wavelength  (λ_max_ nm) | Absorbance^*^ | | | |
| --- | --- | --- | --- | --- |
|  | **CC1** | **CC2** | **CC3** | **CEOC6** |
| 290 | 1.909±0.04 | 1.912±0.02 | 1.969±0.10 | 0.799±0.12 |
| 295 | 1.809±0.02 | 1.781±0.01 | 1.874±0.11 | 0.890±0.11 |
| 300 | 0.709±0.04 | 1.978±0.03 | 2.962±0.10 | 0.980±0.12 |
| 305 | 0.800±0.05 | 0.801±0.02 | 1.679±0.10 | 0.797±0.13 |
| 310 | 0.999±0.02 | 0.898±0.01 | 0.963±0.12 | 0.992±0.11 |
| 315 | 0.999±0.03 | 0.999±0.02 | 0.905±0.11 | 0.888±0.13 |
| 320 | 0.909±0.03 | 0.678±0.01 | 0.817±0.11 | 0.853±0.11 |
| Calculated SPF | 9.28 | 12.68 | 18.54 | 9.02 |

^*^ n=3±SD
